# Expression of Key Structural Genes of the Phenylpropanoid Pathway Associated with Catechin Epimerization in Tea Cultivars

**DOI:** 10.3389/fpls.2017.00702

**Published:** 2017-05-03

**Authors:** Changsong Chen, Kang Wei, Liyuan Wang, Li Ruan, Hailin Li, Xiaogui Zhou, Zhenghe Lin, Ruiyang Shan, Hao Cheng

**Affiliations:** ^1^Tea Research Institute, Fujian Academy of Agricultural SciencesFu’an, China; ^2^Key Laboratory of Tea Plant Biology and Resources Utilization, Ministry of Agriculture, National Center for Tea Improvement, Tea Research Institute, Chinese Academy of Agricultural SciencesHangzhou, China

**Keywords:** *Camellia sinensis*, catechin epimerization, anthocyanidin synthase, anthocyanidin reductase, gallocatechin gallate

## Abstract

Catechin epimerization is an important factor affecting tea catechin compositions and thereby tea quality. However, a lack of tea germplasms with high non-epicatechins limits relative research. Here, a tea cultivar Y510 with high non-epicatechins was firstly reported and used for catechin and RNA sequencing (RNA-Seq) analysis. Results showed that the (-)-gallocatechin gallate and (+)-catechin (C) contents in Y510 were at least 136 and 6 times higher than those in Fudingdabaicha and 0306I, but the epicatechins (-)-epigallocatechin and (-)-epicatechin (EC) were significantly lower. Eleven unigenes potentially involved in catechin epimerization were identified by RNA-Seq analysis. Based on a combination of catechin and gene expression analysis, it was hypothesized that two anthocyanidin reductase genes (*CsANR1*, *CsANR2*) and an anthocyanidin synthase gene (*CsANS*) are the key genes affecting catechin epimerization in tea. Non-epicatechin formations were hypothesized to be mainly influenced by the expression ratio of *CsANR2* to *CsANR1* and the expression of *CsANS*. Overexpression of *CsANS* in an Arabidopsis mutant *tds4-2* led to a significant increase of EC accumulation in seeds, revealing *CsANS* is important for catechin epimerization. These results shed new light on breeding tea cultivars with special catechin compositions.

## Introduction

Tea (*Camellia sinensis*) is a worldwide popular beverage and its consumption is related to many health benefits, such as reducing the risks of cancers (Fujiki et al., 2002), cardiovascular disease (Tijburg et al., 1997), preventing obesity-related disorder (Wolfram et al., 2006) and other oxidative stress-related diseases (Song et al., 2002; Lambert et al., 2005). Those beneficial effects are mainly attributed to the high accumulation of catechins, which occupy 6–16% of dry tea leaves (Cheng et al., 2015). Tea catechins mainly include EGCG, ECG, EGC, EC and C. Generally, EGCG is the most abundant component, accounting for about half of the total catechins (Wei et al., 2011). It has the potential to affect multiple biological pathways involved in cancer prevention or cholesterol metabolism and thereby considered as the most biologically active components in normal green tea (Bursill and Roach, 2006; Chen et al., 2011; Du et al., 2012). While, its ester GCG is barely present in normal tea. For example, Jin et al. (2014) investigated the catechin contents of 403 accessions of representative tea germplasms and found the average GCG content was only about 1.5 mg/g, which was the lowest among seven detected catechin components. However, it is of great interest to scientists, as many studies showed that GCG is more effective to inhibit cholesterol absorption than EGCG (Ikeda et al., 2004). Recent study also showed that GCG has a positive effect on bone metabolism (Ko et al., 2009). Furthermore, GCG is significantly less bitter and astringent compared with EGCG, which indicates that high GCG could improve the taste quality of tea (Scharbert and Hofmann, 2005). These beneficial effects suggest that identification of tea germplasm with high GCG content and exploring its potential mechanism will be valuable for tea breeding.

Catechins are synthesized by the phenylpropanoid and flavonoid biosynthetic pathway (Wei et al., 2015). Recent studies found that both accumulation and composition of catechins were highly correlated with the expression levels of relative biosynthetic genes (Rani et al., 2012; Jiang et al., 2013; Xiong et al., 2013; Wei et al., 2015). For example, Rani et al. (2012) studied the catechin contents and relative biosynthetic gene expressions in tea plants under different conditions and found that chalcone isomerase (CsCHI), flavanone 3-hydroxylase (CsF3H), dihydroflavonol 4-reductase (CsDFR), anthocyanidin synthase (CsANS) and anthocyanidin reductase (CsANR) were amongst the critical regulatory genes in regulating catechin contents. We previously also identified F3′H and F3′5′H are key enzymes involved in the formation of dihydroxylated and trihydroxylated catechins by transcriptome analysis (Wei et al., 2015). Catechin epimerization, mainly affected by the enzymatic catalysis of LAR, ANS and ANR, plays a key role in determining epi (EGCG, ECG, EGC and EC) and non-epi (GCG, CG, GC and C) catechin compositions (Punyasiri et al., 2004; Pang et al., 2013; Wu et al., 2014). LAR catalyzes the conversion of leucocyanidin and leucodelphinidin to the corresponding C and GC (Tanner et al., 2003). ANS converts leucocyanidin and leucodelphinidin to anthocyanidins (Wilmouth et al., 2002). While ANR is participated in the production of the flavan-3-ol monomers (such as EC and EGC from anthocyanidins) (Ashihara et al., 2010). However, a lack of tea germplasms with high non-epi-forms of catechins prevents the identification of key genes affecting catechin epimerization.

Recently, a tea cultivar Y510 with extremely high GCG content was newly bred by our institute. It is a natural hybrid offspring of the cultivar Lechang Baimaocha and suitable to make white tea. Understanding its catechin composition and relative mechanism will largely facilitate the usage of this new cultivar. Moreover, the development of RNA sequencing (RNA-Seq) technology provided us a useful method to identify key genes in non-reference species like tea plant (Paul et al., 2014; Wei et al., 2015; Wu et al., 2016). A combination of metabolome and RNA-Seq analysis can offer precise information about gene-to metabolite networks (Liberman et al., 2012).

In this study, tea samples (one leaf and a bud stage) from Y510 and normal tea cultivars Fudingdabaicha and 0306I were used for catechin and RNA-seq analysis (NCBI BioProject Accession: PRJNA344817, PRJNA344819 and PRJNA344821). Furthermore, a key *CsANS* gene was introduced into an Arabidopsis ANS knockout mutant (*tds4-2*) to verify its potential role in catechin epimerization. The purposes of this work were to identify key genes affecting catechin epimerization in *C. sinensis*.

## Materials and Methods

### Plant Materials

Fresh materials (one leaf and a bud) of the first flush shoots of three tea cultivars (Fudingdabaicha, 0306I and Y510) grown in the experimental tea garden of Tea Research Institute, Fujian Academy of Agricultural Sciences in Fu’an, China (119°35′E, 27°10′N) were collected in Match, 2015. Each sample contained fresh materials harvested from more than 100 tea plants. The collected samples were stored at -80°C. Parts of the tea samples were dried by a freeze dryer (Labconco Stoppering Tray Dryer, Labconco Inc., Kansas City, MO, USA) at -5°C for 72 h, then subject to HPLC analysis of catechins. The rest samples were subjected to RNA-Seq analysis.

### Catechin Analysis

The standard chemicals, including GCG, C, EC, EGC, EGCG and ECG were purchased from Sigma Chemical Company (St. Louis, MO, USA). The extraction method and HPLC analysis of catechin contents in each tea samples were exactly according to the method described by Wei et al. (2015). The total catechin contents were calculated as the sum of six individual catechins. Three replications were taken for each sample. Data were expressed as means ±SD and evaluated by one-way ANOVA followed by Duncan’s multiple comparison test at *p* < 0.05. SPSS 16.0 software was used for statistical analysis.

### RNA-Seq Analysis

RNA isolation of tea samples (Fudingdabaicha, 0306I and Y510), RNA-Seq, *de novo* assembly and functional annotation were performed according to the method described by Wei et al. (2015). Illumina HiSeq2500 genome analyzer was utilized for RNA-Seq. FPKM were used to show the transcript abundances by software RSEM. Major genes annotated to LAR, ANS and ANR were identified for further analysis.

### Quantitative Real Time PCR Analysis

To verify the RNA-Seq results, six major genes, including three LAR genes, an ANS gene and two ANR genes potentially involved in catechin epimerization were selected for qRT-PCR analysis. Primers were designed using Primer 3 software (**Supplementary Table S1**). RNA isolation by a modified CTAB method and qRT-PCR assays were performed as described by Wei et al. (2016). GAPDH was utilized as internal control to calculate and normalize relative expression of candidate genes in each tea samples. Four groups of fresh materials were randomly selected from each tea sample for RNA isolation separately. Each group was considered as a biological replicate. The data were expressed as the means ± SD from four independent biological replicates.

### Complementation Analysis and Culture

Full length cDNA of *CsANS* was cloned into binary vector pCAMBIA1300 containing the 35S promoter. The vector was electroporated into Agrobacterium strains GV3101 to infect *Arabidopsis thaliana* (L.) *tds4-2* mutant (Salk_028793, an Arabidopsis *ANS* knockout mutant barely accumulates anthocyanins, Abrahams et al., 2003) with the floral dip method (Clough and Bent, 1998). Phenotypes of the T1 seedlings were characterized at the seedling stage as described by Kitamura et al. (2012). Two T2 lines (*tds4-OE1* and *tds4-OE2*) with the highest *CsANS* transcript levels were chosen for further analysis.

All Arabidopsis lines (*tds4-2, tds4-OE1* and *tds4-OE2*) were grew in a growth chamber under a 16/8 h light/dark photoperiod at 24°C. Mature seeds were harvested and subjected to catechin analysis according to the method described by Wei et al. (2015).

## Results

### The Variation of Catechin Contents among Cultivars

**FIGURE 1:**
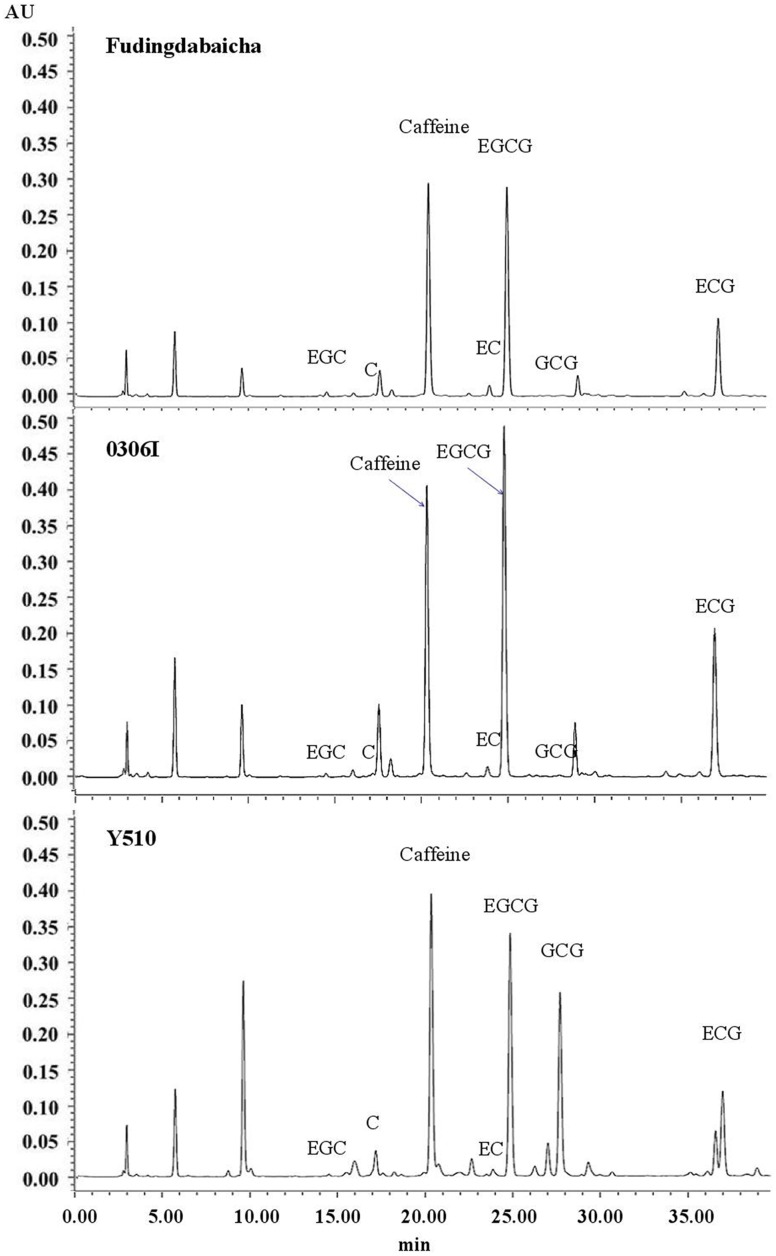
**HPLC chromatogram of catechins of three tea cultivars: Fudingdabaicha, 0306I and Y510**.

Typical chromatograms of tea catechins separated from cv. Fudingdabaicha, 0306I and Y510 are present in **Figure 1**. An extremely high GCG peak was observed in Y510. While the GCG peaks of Fudingdabaicha and 0306I were much lower. The HPLC profile of Y510 is clearly different from those of the other two cultivars.

The means and SD of six individual catechins and total catechin contents in three cultivars are shown in **Table 1**. The total catechin content of Y510 was 1.1 and 1.8 folds higher than those of 0306I and Fudingdabaicha, indicating Y510 had a more active biosynthesis of catechins. In term of individual catechins, the GCG and C contents in Y510 reached 54.5 and 16.8 mg/g, respectively, which were 272.5 and 6.2 times higher than those in 0306I, and 136.3 and 12.0 folds higher than those in Fudingdabaicha. By contrast, the epicatechins EGC and EC were significantly lower in Y510 compared with 0306I and Fudingdabaicha. Moreover, the percentage of non-epi catechins to the total catechins of Y510 reached 41.5%, which was much higher than those of Fudingdabaicha (1.8%) and 0306I (1.9%). All these results suggest Y510 has an abnormal catechin epimerization, which would be useful to explore the underlying mechanism.

**Table 1:** Mean and standard deviation (SD) of catechins in three tea cultivars.

	Mean ±*SD* (mg/g)						
Cultivars	EGC	C	EC	EGCG	GCG	ECG	Total Catechins
Fudingdabaicha	7.2 ± 0.7a	1.4 ± 0.2c	6.5 ± 0.3a	61.7 ± 6.7b	0.4 ± 0.0b	20.1 ± 2.2b	97.3 ± 9.8c
0306I	5.7 ± 0.4b	2.7 ± 0.1b	6.0 ± 0.3a	100.2 ± 5.7a	0.2 ± 0.0b	37.9 ± 2.2a	152.7 ± 8.6b
Y510	3.9 ± 0.2c	16.8 ± 0.4a	4.9 ± 0.2b	69.6 ± 1.6b	54.5 ± 1.3a	22.0 ± 0.5b	171.7 ± 4.3a

*The different letters after data within a column represent significant difference at 95% probability level*.

### RNA-Seq Analysis

To further explore potential genes associating with the abnormal catechin epimerization in Y510, tea samples of the three tea cultivars (Fudingdabaicha, 0306I and Y510) were subjected to RNA-Seq analysis. Totally 127.7 million 125-bp paired-end reads were obtained, with more than 127.5 million clean reads (**Table 2**). Data of Fudingdabaicha, 0306I and Y510 were deposited in the NCBI with accession number of PRJNA344817, PRJNA344819 and PRJNA344821. Subsequently, the clean reads were assembled into 108,926 unigenes by software Trinity, with an average length of 700 bp and N50 length of 1046 bp (**Supplementary Table S2**).

**Table 2:** Summary for RNA-Seq datasets of *C. sinensis*.

Cultivars	Raw reads (million)	Raw bases (Gb)	Clean reads (million)	Clean bases (Gb)	Clean data rate (%)
Fudingdabaicha	39.87	4.98	39.84	4.96	99
0306I	43.35	5.42	43.31	5.39	99
Y510	44.45	5.56	44.41	5.53	99

Totally 36.4% of the unigenes (39,606) were annotated by four major public databases (GO, KEGG, Pfam and NR databases) with an *E*-value threshold of 10^-5^ (**Supplementary Table S3**). Consistent with previous findings, it was found 99.2% of the annotated unigenes were annotated to the NR database, indicating NR database is the most important public database for gene annotation in *C. sinensis* (Wei et al., 2015, 2016).

As gene expressions are mainly regulated by transcription factors, differently expressed genes (the expression differences between Y510 and the other cultivars >2) annotating to transcription factors were identified, with 139 up-regulated and 41 down-regulated in Y510 (**Supplementary Table S4**). Those transcription factors belong to multiple families, including Zinc finger (24), U-box (24), basic helix-loop-helix (22), F-box (16), WRKY (13) and MYB (9). Among them, a Zinc finger gene (Unigene102034) highly similar to TRANSPARENT TESTA 1 (AT1G34790) and a WRKY family transcription factor (Unigene003160) similar to TRANSPARENT TESTA GLABRA 2 (AT2G37260) were identified to be up-regulated in Y510. Both AT1G34790 and AT2G37260 play key roles in flavonoid biosynthesis, suggesting their homolog genes might affect catechin epimerization in tea plants (Johnson et al., 2002; Sagasser et al., 2002).

**FIGURE 2:**
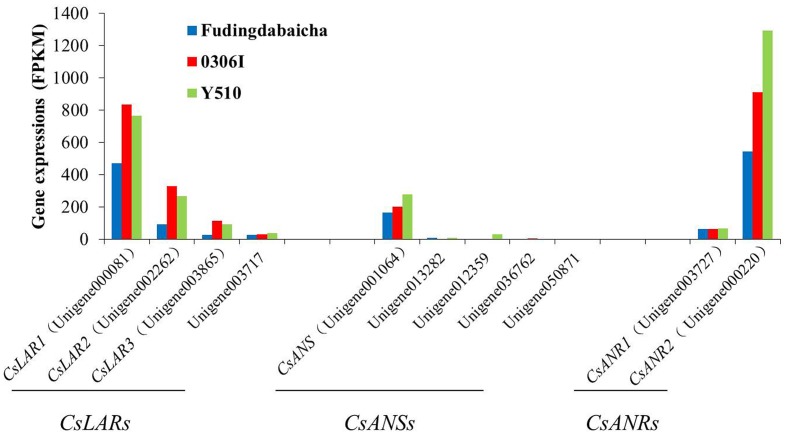
**The expression of candidate genes involved in catechin epimerization (including four *LAR* unigenes, five *ANS* unigenes and two *ANR* unigenes) in Fudingdabaicha, 0306I and Y510 by RNA-Seq analysis**.

On the other hand, transcription factors regulate the expressions of functional genes, whose changes finally affect the traits. Therefore, it is also necessary to understand the expression changes of those functional genes. Eleven unigenes (including four *LAR* unigenes, five *ANS* unigenes and two *ANR* unigenes) potentially involved in catechin epimerization were identified according to the gene annotation. Results showed that the expressions of three *LAR* unigenes [unigene000081 (*CsLAR1*), unigene002262 (*CsLAR2*) and unigene003865 (*CsLAR3*)], an *ANS* unigenes [unigene001064 (*CsANS*)] and two *ANR* unigenes [unigene003727 (*CsANR1*) and unigene000220 (*CsANR2*)] showed higher expression levels than the rest unigenes, indicating they might be more important and thus, chosen as candidate genes for subsequent study (**Figure 2**).

Blast analysis showed that *CsLAR1*, *CsLAR2* and *CsLAR3* were 99, 82, and 74% identity to the *CsLAR* gene (GenBank accession no. GU992401). While *CsANS, CsANR1* and *CsANR2* were more than 99% identity to the *CsANS* (GenBank accession no. KR902746), *CsANR1* (GenBank accession no. GU992402) and *CsANR2* (GenBank accession no. GU992400) respectively, suggesting they might be the same genes in different cultivars. As LAR was considered as the key enzyme catalyzing the formation of non-epicatechins, the expressions of relative genes were paid more attention (Punyasiri et al., 2004; Pang et al., 2013; Wu et al., 2014). However, it is interesting that the expressions of three key *LAR* unigenes in Y510 showed no clear differences as compared with the other two cultivars. By contrast, *CsANS* and *CsANR2* exhibited clearly higher expressions in Y510 than those in Fudingdabaicha and 0306I. These results suggest *CsANS* and *CsANR2* might play more important roles in affecting catechin epimerization.

### Verification of Key Genes by qRT-PCR

**FIGURE 3:**
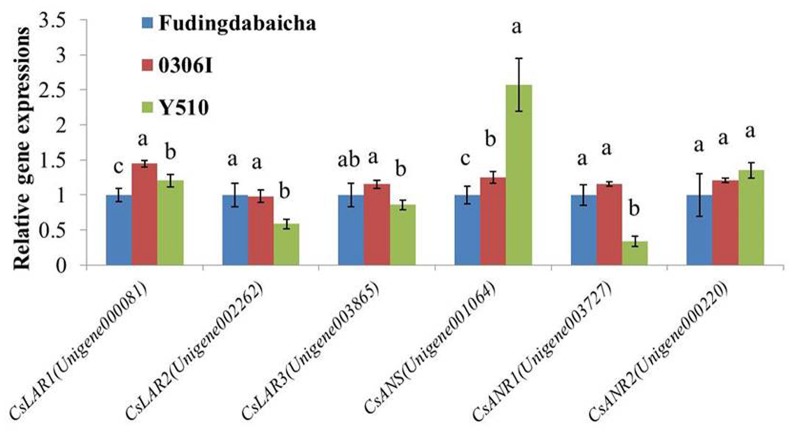
**Relative expressions of six candidate genes in Fudingdabaicha, 0306I and Y510 determined by qRT-PCR**. Data of real time PCR analysis are the means ± SD (*n* = 3). Gene expressions in Fudingdabaicha were taken as 1.0 and their relative expressions in other samples were calculated accordingly. The different letters on the columns represent significant difference at 95% probability level.

To verify the RNA-Seq results, qRT-PCR was performed to analyze the expressions of the six key genes (*CsLAR1*, *CsLAR2, CsLAR3, CsANS, CsANR1* and *CsANR2*) in three tea cultivars (**Figure 3**). None of the *CsLAR* genes showed obviously higher expression levels in Y510, which was consistent with the RNA-Seq results and suggests catechin epimerization in tea plants might not be attributed to *CsLARs.* In contrast, significantly higher expression of *CsANS* and lower expression of *CsANR1* were observed in Y510 as compared to Fudingdabaicha and 0306I. Moreover, *CsANR2* showed a slightly but not significantly higher expression in Y510. These results suggest that the expressions of three genes (*CsANR1*, *CsANR2* and *CsANS*) might play key roles in catechin epimerization in tea plants, which are worth further studies.

### Functional Characterization of *CsANS* in Transgenic Plants

As the functions of *CsANR1* (GU992402), *CsANR2* (GU992400) and *CsLAR* (GU992401) had already been characterized in the transgenic tobacco overproducing anthocyanin, it is also necessary to characterize the function of *CsANS* in catechin epimerization in transgenic plants (Pang et al., 2013). Thus, the cauliflower mosaic virus (CaMV) 35S promoter-driven open reading frame (ORF) of *CsANS* was introduced into *tds4-2* (Salk_028793), an Arabidopsis *ANS* knockout mutant barely accumulates anthocyanins (Abrahams et al., 2003). Ten transgenic lines were identified from the transformants and two lines with the highest *CsANS* transcript levels (*tds4-OE1* and *tds4-OE2*) were chosen for further analysis.

**FIGURE 4:**
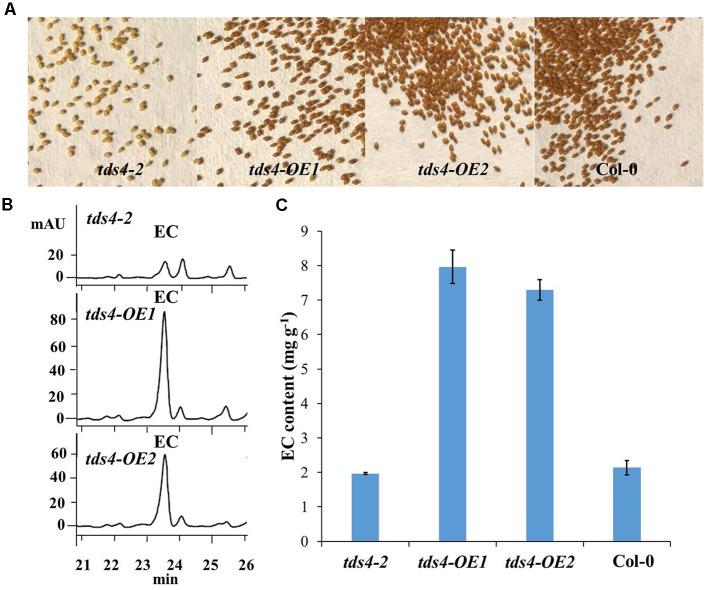
**Phenotypes and EC contents of Arabidopsis seeds of *tds4-2*, transgenic lines *tds4-OE1*, *tds4-OE2* and Col-0**. **(A)** Phenotypes of Arabidopsis seeds of *tds4-2, tds4-OE1*, *tds4-OE2* and Col-0. **(B)** Typical HPLC chromatograms of EC contents in the seeds of *tds4-2, tds4-OE1* and *tds4-OE2*. **(C)** EC contents in Arabidopsis seeds of *tds4-2, tds4-OE1*, *tds4-OE2* and Col-0. Data are means ± SE from three biological replicates.

As EC has already been proved to be available in the seeds of Arabidopsis (Routaboul et al., 2006; Kitamura et al., 2010), the phenotypes and EC contents in seeds of *tds4-OE1* and *tds4-OE2* were compared with those of *tds4-2* (**Figure 4**). A clear *transparent testa* phenotype was observed in *tds4-2* seeds (**Figure 4A**). While seeds of the transgenic lines and wild type (Col-0) showed the deep color, which indicates *CsANS* has a similar function as *AtANS* (AT4G22880) and could rescue the *transparent testa* phenotype of *tds4-2*. Further catechin analysis was performed to check the effect of *CsANS* on EC accumulation in Arabidopsis seeds. Typical HPLC chromatograms showed that the transgenic lines had clearly higher EC peaks than that of *tds4-2* (**Figure 4B**). Furthermore, catechin analysis showed that the EC contents in seeds of *tds4-OE1* and *tds4-OE2* reached 8.0 and 7.3 mg g^-1^, which were 4.0 and 3.7 times higher than those in *tds4-2*, respectively. While EC content in Col-0 was only 2.1 mg g^-1^, which was slightly higher than that in *tds4-2*. These results confirmed that *CsANS* plays an important role in catechin epimerization.

## Discussion

Epimerization of tea catechins plays a key role in determining catechin compositions. Generally, the epicatechin contents in tea are much higher than their esters. For example, Jin et al. (2014) found epicatechins accounted for more than 95% of the total catechins in most tea germplasms. Here, a tea cultivar Y510 with extremely high GCG content was firstly reported. The percentage of non-epi catechins to the total catechins of Y510 reached 41.5%, much higher than the rest cultivars, which offers a good genetic resource to facilitate our understanding of *in vivo* epimerization of tea catechins.

**FIGURE 5:**
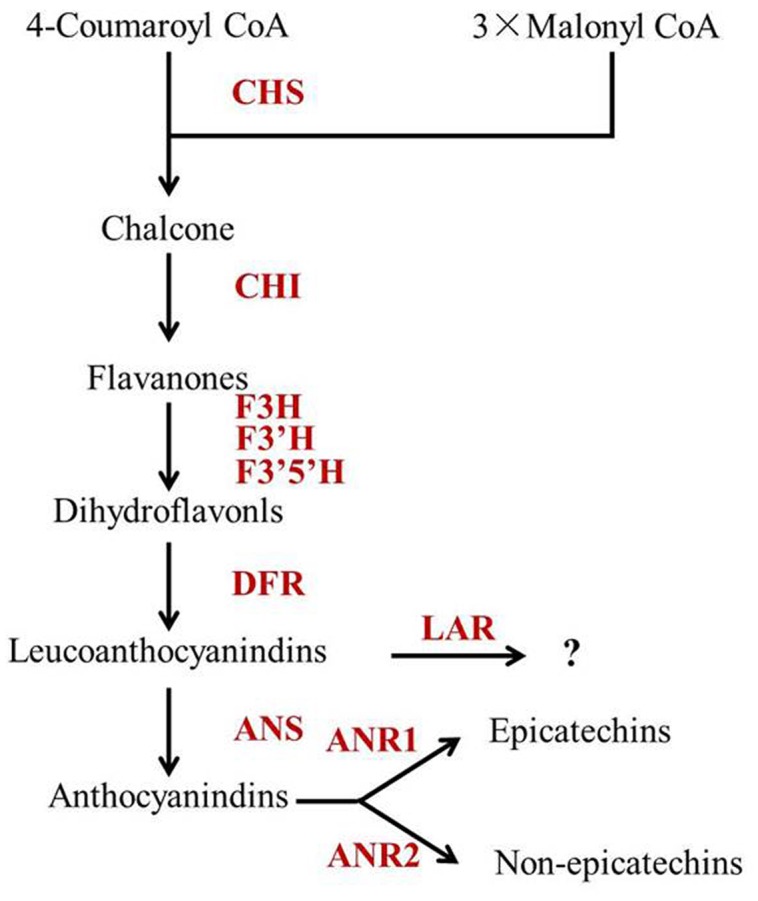
**The proposed catechin biosynthetic pathway in *Camellia sinensis***. Abbreviations of enzymes are as follows: CHS, chalcone synthase (EC 2.3.1.74); CHI, chalcone isomerase (EC 5.5.1.6); F3H, flavanone 3-hydroxylase (EC 1.14.11.9); F3′5′H, flavonoid 3′,5′-hydroxylase (EC 1.14.13.88); F3′H, flavonoid 3′-hydroxylase (EC 1.14.13.21); DFR, dihydroflavonol 4-reductase (EC 1.1.1.219); ANS, anthocyanidin synthase (EC 1.14.11.19); ANR, anthocyanidin reductase (EC 1.3.1.77); LAR, leucoanthocyanidin reductase (EC 1.17.1.3).

Leucoanthocyanidin reductase, ANS and ANR are downstream enzymes in the flavonoid biosynthetic pathway, which play key roles in determining individual catechins (**Figure 5**). Pang et al. (2013) reported that CsLAR (GU992401), which shares more than 99% amino acid identity with the CsLAR1 identified in this study, *in vitro* catalyzes the conversion of leucocyanidin to C. Therefore, CsLAR was hypothesized as the key enzyme affecting non-epicatechin formation in previous studies (Ashihara et al., 2010; Wei et al., 2011). However, overexpression of *CsLAR* in tobacco overproducing anthocyanin led to the accumulation of higher levels of EC and ECG than C (Pang et al., 2013). Likewise, the expression of LARs from other species in transgenic plants did not produce detectable levels of C *in vivo* (Bogs et al., 2005; Pang et al., 2007; Paolocci et al., 2007). Furthermore, none of the previous correlation analysis showed a direct correlation between the expressions of *CsLAR* genes and non-epicatechins in tea plants (Ashihara et al., 2010; Xiong et al., 2013; Zhang et al., 2016). These studies suggest that the *in vivo* function of *CsLAR* in plants might be different from that observed in *in vitro* assays. Our study showed much higher accumulation of non-epicatechins but no obviously higher expressions of *CsLARs* in Y510 as compared with those in normal tea cultivars, which was consistent with previous studies and indicates that *CsLARs* might not be the key factors influencing the *in vivo* formation of non-epicatechins in tea.

On the other hand, *in vitro* assays showed that both CsANR1 (GU992402) and CsANR2 (GU992400) were able to convert cyanidin to a mixture of EC and C, and delphinidin to a mixture of EGC and GC, demonstrating both enzymes also participate in the formation of non-epicatechins in tea (Pang et al., 2013). Moreover, both enzymes showed different proportions of products, with CsANR1 preferentially formed epicatechins, while CsANR2 preferentially produced non-epicatechins. However, as overexpressing either *CsANR1* or *CsANR2* in tobacco did not result in clear increases of catechins, it is still lack of *in vivo* evidences to support their roles in catechin formation (Pang et al., 2013). Our results showed a significantly higher expression ratio of *CsANR2* to *CsANR1* in Y510 than those in the other cultivars according to both RNA-Seq and qRT-PCR analysis, which firstly provided an *in vivo* evidence that the expression ratio of *CsANR2* to *CsANR1* may directly affect catechin epimerization in tea (**Figures 2**, **3**).

Moreover, ANS was previously hypothesized to be a negative regulator of non-epicatechins, as it might compete for substrates with LAR (Ashihara et al., 2010; Wei et al., 2011). However, our results showed a positive correlation between the expression of *CsANS* and non-epicatechins (C and GCG). Similar results were also found in many other correlation analysis between catechin accumulation and gene expressions in tea plants. For example, Liu et al. (2015) reported that the expression of *CsANS* was positively correlated with C, but negatively correlated with EC and EGC in spring tea. Zhang et al. (2016) also studied the correlations between the expressions of flavonoid synthetic genes and catechin accumulations in tea leaves of different stages and found *CsANS* was positively correlated with C. The negative correlation between *CsANS* and catechin epimerization may be attributed to the key roles of *CsANRs* in non-epicatechin formation, as *CsANS* provides substrates for *CsANRs* (Martens et al., 2010). To prove this, *CsANS* was introduced into the Arabidopsis mutant *tds4-2* in this study and the EC contents in seeds were largely increased in the transgenic lines (**Figure 4C**). This indicates *CsANS* is closely associated with catechin epimerization. Furthermore, overexpression of *CsANS* led to a significant increase of EC but not C, which might be attributed to the function of its downstream gene *AtANR*. Xie et al. (2003) reported that AtANR preferentially produces EC, which is similar to the function of CsANR1 (Pang et al., 2013). Therefore, the non-epicatechin formation in tea plant was hypothesized to be mainly affected by the expression of *CsANS* as well as the expression ratio of *CsANR2* to *CsANR1* (**Figure 5**).

## Conclusion

In summary, a tea cultivar Y510 with high C and GCG contents was firstly reported and used to study catechin epimerization. Based on a combination of catechin and gene expression analysis, it was hypothesized that *CsANR1*, *CsANR2* and *CsANS* are the key genes affecting catechin epimerization in tea. Non-epicatechin formations may be mainly influenced by the expression ratio of *CsANR2* to *CsANR1* and the expression of *CsANS* (**Figure 5**), which shed new light on tea breeding, especially for those with special catechin composition.

## Availability of Data and Materials

Illumina sequence data are available from the Short Read Archive under accession SRP090603 (Fudingdabaicha), SRP090604 (0306I) and SRP090606 (Y510). The NCBI BioProject Accession for each cultivar are PRJNA344817 (Fudingdabaicha), http://www.ncbi.nlm.nih.gov/bioproject/344817; PRJNA344819 (0306I), http://www.ncbi.nlm.nih.gov/bioproject/344819; and PRJNA344821 (Y510) http://www.ncbi.nlm.nih.gov/bioproject/344821.

## Author Contributions

Conceived and designed the experiments: CC, KW, and HC. Performed the experiments: CC, KW, LW, LR, HL, XZ, ZL, and RS. Analyzed the data: CC, KW, and HC. Contributed reagents/materials/analysis tools: KW and HC. Contributed to the writing of the manuscript: CC and KW. All authors have read and approved this manuscript.

## Conflict of Interest Statement

The authors declare that the research was conducted in the absence of any commercial or financial relationships that could be construed as a potential conflict of interest.

## Supplementary Material

TABLE S1 **Primers used for quantitative real time PCR analysis**.

TABLE S2 **Length distribution of assembled unigenes**.

TABLE S3 **Summary for the BLASTx results of *C. sinensis* transcriptome against public databases**.

TABLE S4 **List of differentially expressed transcription factors in Y510 by RNA-Seq analysis**.

## Abbreviations

ANOVA: analysis of variance
ANR: anthocyanidin reductase
ANS: anthocyanidin synthase
C: (+)-catechin
CG: (-)-catechin gallate
CHI: chalcone isomerase
DFR: dihydroflavonol 4-reductase
EC: (-)-epicatechin
ECG: (-)-epicatechingallate
EGC: (-)-epigallocatechin
EGCG: (-)-epigallocatechin gallate
F3′5′H: flavonoid 3′,5′-hydroxylase
F3′H: flavonoid 3′-hydroxylase
F3H: flavanone 3-hydroxylase
FPKM: Fragments per kilobase of transcript per million reads
GC: (-)-gallocatechin
GCG: (-)-gallocatechin gallate
LAR: leucoanthocyanidin reductase
NCBI: National Center for Biotechnology Information
SD: standard deviations.
