# Supplementary material for: Expression of Key Structural Genes of the Phenylpropanoid Pathway Associated with Catechin Epimerization in Tea Cultivars
Source: Front Plant Sci. 2017 May 3;8:702. doi: 10.3389/fpls.2017.00702 (PMC5413559; doi:10.3389/fpls.2017.00702)
Supplement: TABLE S1 — Primers used for quantitative real time PCR analysis. [file Table_1.DOC]

**Table S1. Primers used for quantitative real time PCR analysis**

| **Gene no.** | **Functional annotation** | **Primer sequence(5’-3’)** |
| --- | --- | --- |
| Unigene000081 | *CsLAR1* | F: ATCCCACAAAGTGTCGTTGC |
|  |  | R: GCAGTGTTTCCATCCGTCTC |
| Unigene002262 | *CsLAR2* | F: TGGCTTTGACAATGGTGAGC |
|  |  | R: TTCTTCTCGTCCGCTCACTT |
| Unigene003865 | *CsLAR3* | F: CCACATTCAAGCACACATTGG |
|  |  | R: CGTTGCATCATTCACACACG |
| Unigene001064 | *CsANS* | F: GTAGGAGGCATGGAAGAGCT |
|  |  | R: TAATGGAGTTGGGGACGCAT |
| Unigene003727 | *CsANR1* | F: CAGTGTTCCAGAGCTTGCAA |
|  |  | R: TTCAGTTCTGCAAAAGCCCC |
| Unigene000220 | *CsANR2* | F: TGAGAAAGGCTATGCGGTCA |
|  |  | R: TGGATCCTCGGAAGCAAAGT |
| GE651107 | *GAPDH* | F: TTGGCATCGTTGAGGGTCT |
|  |  | R: CAGTGGGAACACGGAAAGC |
