# Supplementary material for: Expression of Key Structural Genes of the Phenylpropanoid Pathway Associated with Catechin Epimerization in Tea Cultivars
Source: Front Plant Sci. 2017 May 3;8:702. doi: 10.3389/fpls.2017.00702 (PMC5413559; doi:10.3389/fpls.2017.00702)
Supplement: TABLE S2 — Length distribution of assembled unigenes. [file Table_2.DOC]

**Table S2. Length distribution of assembled unigenes.**

| **Unigenes Length (bp)** | **Number of sequences** | **Percentage** |
| --- | --- | --- |
| 200-300 | 33879 | 31.10% |
| 300-500 | 31086 | 28.54% |
| 500-1000 | 24565 | 22.55% |
| 1000-2000 | 12391 | 11.38% |
| 2000+ | 6977 | 6.41% |
| Total number | 108926 |  |
| Average length | 700 bp |  |
| N50 length | 1046 bp |  |
