# Supplementary material for: Expression of Key Structural Genes of the Phenylpropanoid Pathway Associated with Catechin Epimerization in Tea Cultivars
Source: Front Plant Sci. 2017 May 3;8:702. doi: 10.3389/fpls.2017.00702 (PMC5413559; doi:10.3389/fpls.2017.00702)
Supplement: TABLE S3 — Summary for the BLASTx results of C. sinensis transcriptome against public databases. [file Table_3.DOC]

**Table S3. Summary for the BLASTx results of *C. sinensis* transcriptome against public databases**

| **Annotation database** | **Annotated Number** |
| --- | --- |
| GO Annotation | 12913 |
| KEGG Annotation | 11157 |
| Pfam Annotation | 19605 |
| NR Annotation | 39284 |
| All Annotated unigenes | 39606 |
